# Heterogeneous Types of miRNA-Disease Associations Stratified by Multi-Layer Network Embedding and Prediction

**DOI:** 10.3390/biomedicines9091152

**Published:** 2021-09-03

**Authors:** Dong-Ling Yu, Zu-Guo Yu, Guo-Sheng Han, Jinyan Li, Vo Anh

**Affiliations:** 1 Key Laboratory of Intelligent Computing and Information Processing of Ministry of Education, Xiangtan University, Xiangtan 411105, China; 201931000105@smail.xtu.edu.cn (D.-L.Y.); hangs@xtu.edu.cn (G.-S.H.); 2 Hunan Key Laboratory for Computation and Simulation in Science and Engineering, Xiangtan University, Xiangtan 411105, China; 3 Data Science Institute, University of Technology Sydney, Broadway, NSW 2007, Australia; 4 Faculty of Science, Engineering and Technology, Swinburne University of Technology, Hawthorn, VIC 3122, Australia; vanh@swin.edu.au

**Keywords:** miRNA-disease, heterogeneous association types, attributed multi-layer heterogeneous network embedding, Node2vec

## Abstract

Abnormal miRNA functions are widely involved in many diseases recorded in the database of experimentally supported human miRNA-disease associations (HMDD). Some of the associations are complicated: There can be up to five heterogeneous association types of miRNA with the same disease, including genetics type, epigenetics type, circulating miRNAs type, miRNA tissue expression type and miRNA-target interaction type. When one type of association is known for an miRNA-disease pair, it is important to predict any other types of the association for a better understanding of the disease mechanism. It is even more important to reveal associations for currently unassociated miRNAs and diseases. Methods have been recently proposed to make predictions on the association types of miRNA-disease pairs through restricted Boltzman machines, label propagation theories and tensor completion algorithms. None of them has exploited the non-linear characteristics in the miRNA-disease association network to improve the performance. We propose to use attributed multi-layer heterogeneous network embedding to learn the latent representations of miRNAs and diseases from each association type and then to predict the existence of the association type for all the miRNA-disease pairs. The performance of our method is compared with two newest methods via 10-fold cross-validation on the database HMDD v3.2 to demonstrate the superior prediction achieved by our method under different settings. Moreover, our real predictions made beyond the HMDD database can be all validated by NCBI literatures, confirming that our method is capable of accurately predicting new associations of miRNAs with diseases and their association types as well.

## 1. Introduction

MicroRNAs (miRNAs) are a class of non-coding single-stranded RNA molecules with a length of about 22 nucleotides encoded from endogenous genes. It has been found that the abnormally high or low expressions of miRNAs are closely related to disease progression and development, such as tumor progression [1,2]. Therefore, miRNAs can be used as biomarkers for disease diagnosis or drug targets in treatment design [3,4]. These associations between miRNAs and diseases are sometimes very complicated with up to five heterogeneous types induced by genetics, epigenetics, circulating miRNAs, miRNA tissue expression and miRNA-target interactions.

An early version of the database HMDD (v2.0) [5] stratifies miRNA-disease associations into four types (denoted as Type-1, Type-2, Type-3 and Type-4 here). Type-1 is defined as a special association induced by genetics. It is mainly confirmed by GWAS analysis, gene knockout and gene overexpression. For example, two SNPs of rs41275794 and rs12976445 in pri-miR-125a change the expression of mature miR-125a and are associated with recurrent abortion [6]; the deletion of miR-15a increases the risk of Lymphoma [7]; the proliferation, invasion and metastasis of HCT116 cells can be inhibited by overexpression of miR-34a in colon cancer [8]. Type-2 is a special association induced by epigenetics change. Mutations of epigenetic components are common in diseases [9]. It has been proved that miRNAs can serve on modulators of epigenetic through targeting key enzymes that are responsible for epigenetic responses, and the expression of miRNAs can be regulated by epigenetic mechanism [10]. The reciprocal interaction between epigenetic and miRNA regulation forms an epigenetic-miRNA feedback loop. Type-3 association is specified from circulation assays. Since circulating miRNAs are always remarkably stable in plasma and serum under harsh conditions, they can be used as novel biomarkers for diagnosis and prognosis [11,12]. Circulating miRNAs can also be selectively targeted for secretion in one cell and absorbed by a distant target cell to regulate gene expression [13]. Type-4 can be identified through miRNA-target interactions, including miRNA-mRNA interactions and miRNA-lncRNA interactions, as well as feedback loops between miRNAs and transcription factors. For instance, miR-106a targeting MCL1 to inhibit cisplatin resistance of A2780 in ovarian cancer cells [14] implies a Type-4 association between miR-106a and ovarian cancer; the feedback loops between miR-124 and TGF-β pathways play an important role in metastasis of non-small cell lung cancer [15]. Recently, the HMDD database was updated to version v3.2 [16] and added a newly specified classification of Type-5 on the evidence and data from miRNA tissue expression assays. MiRNAs are present in a variety of human tissues, and the differential expression of miRNA and the deregulation expression of miRNA in tissues have been found to be related to the disease. For example, it has been found that six miRNAs, miR-31, miR-34a, miR-181a, miR-181b, miR-193a-3p and miR-193b are all upregulated, but other the four miRNAs miR-221, miR-222, miR-484 and miR-502-3p are all downregulated in pancreatic cancer cells [17]. Moreover, Cui et al. [18] explored a significant positive correlation among body fluid miRNAs and tissue miRNAs. The miRNAs in tissues are highly correlated with miRNAs in male serum and female plasma.

Therefore, the association of miRNA-disease can not only be divided into multiple types according to the association evidences but there are also correlations among the various types. Furthermore, when an miRNA is associated with a disease, the association can be multiple types. For example, the reduced expression level of miR-9 can not only be the prognosis biomarker for Waldenstrom macroglobulinemia but it also causes Waldenstrom macroglobulinemia in humans. The association between miR-223 and inflammation can be proved by genetics, epigenetics or miRNA-target interactions. When one type of association is known for an miRNA-disease pair, it is important to predict any other types of the association for a better understanding of the disease mechanism and for developing accurate treatment for the diseases. It is even more important to reveal associations and association type for currently unassociated miRNAs and diseases. Since confirming associations and association types of miRNA-disease through biological experiments is very time-consuming and costly, it is very necessary to reveal miRNA-disease association and association type through computational methods.

Most of previous algorithms aimed for a binary prediction to observe whether an miRNA-disease pair has an association or not, but these binary prediction algorithms are unable to stratify the multiple heterogeneous types of the association between an miRNA and a disease. To refine the prediction extendable to handle the multiple types of an association, Chen et al. [19] proposed a model (RBMMMDA) based on restricted Boltzman machines (RBMs) where a RBM is constructed for every specific miRNA. All of the diseases are then served as nodes in the visible layer, and the associated state with a specific miRNA acts as the label of the disease node. The association probabilities between a disease and an miRNA are updated by a two-layer undirected graph. RBMMMDA can predict the links of miRNAs associated with diseases as well as multiple association types. However, the visible layers and the hidden layers are not cross connected in RBM, missing the similarity information of the disease network and the miRNA network. Zhang et al. [20] proposed to calculate the Gaussian similarity and semantic similarity for diseases, the function similarity and the Gaussian similarity for miRNAs on each miRNA-disease association type. Then, a model (NLPMMDA) was constructed to uncover unobserved multiple types of miRNA-disease associations. For each association type, they adopted a label propagation algorithm on the integrated disease-disease similarity network and corresponding miRNA-miRNA similarity network. Good performance was achieved by NLPMMDA, whereas the correlation between multi-types of miRNA-disease associations was not taken into consideration. Recently, Huang et al. [21] considered multiple association types between miRNAs and diseases as a tensor and developed a novel tensor completion algorithm (named TDRC) to predict miRNA-disease association types. TDRC treats auxiliary information of miRNA similarities and disease similarities as relational constraints and incorporates it into the optimal objective function. As TDRC takes into account multiple biological information and the correlation between different association types, it performs better than previous models and other traditional tensor completion algorithms in exploiting potential miRNA-disease association types. However, the tensor completion algorithm can only capture linear relationships of disease-miRNA association network. We believe that it would be very effective to predict the miRNA-disease multi-type associations by using the non-linear properties among the disease-miRNA associations, the miRNA similarity network and the disease similarity network.

Recent development in graph embedding or network representation learning have opened the door for exploring non-linear properties in heterogeneous networks. The technique of attributed multi-layer heterogeneous network embedding (GATNE) [22] is a recently emerging graph embedding approach which is able to capture rich attributed information and exploit multiplex topological structures from different node types. In particular, it can project the structural information of the nodes and non-linear relationship of the network into low-dimensional continuous space while preserving inherent properties. Considering that the network of miRNA-disease is complicated, comprising not only multi-typed nodes and associations but also rich similarity information between the nodes of the same type, we are motivated to explore attributed multi-layer heterogeneous network embedding for predictions of multi-typed associations between miRNAs and diseases. We name our method mDLinker.

By our method, the node features of miRNAs and diseases are created through the Node2vec algorithm from their similarity networks. The topology information, the non-linear relationships of miRNA-disease associations and different association types are captured through GATNE from the attributed multi-layer miRNA-disease heterogeneous network. Finally, random forest is trained on the information-rich edges of the network and then applied to predict miRNA-disease association types within the HMDD database or beyond the database. Experimental results show that our method achieved excellent performance in both algorithm comparison and real case studies. Therefore, our algorithm has powerful ability to predict miRNA-disease association and association types. It also has great significance for understanding disease pathology at the molecular level.

## 2. Materials and Methods

### 2.1. Datasets

**Figure 1:**
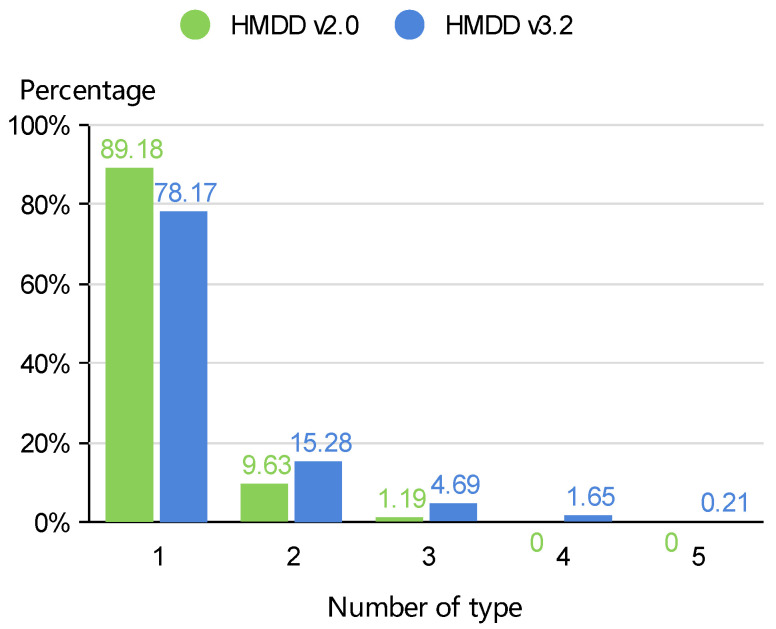
The percentages of miRNA-disease associations containing different numbers of types.

Data records from HMDD, MeSH and miRBase were downloaded for this study. HMDD (http://www.cuilab.cn/hmdd/, accessed on 11 October 2020) is a well-maintained database of miRNA-disease associations. HMDD v2.0 recorded 10,381 miRNA-disease associations between 383 diseases and 577 miRNAs, while the latest version of HMDD v3.2 covers 894 diseases, 1206 miRNAs and 35,548 miRNA-disease associations. The miRBase database (http://www.mirbase.org/, accessed on 15 October 2020) is one of the most important public databases of miRNAs, which provides published miRNA precursor sequences, their annotations, predicted gene targets and so on. The directed acyclic graph description of diseases is obtained from the Medical Subject Heading (MeSH) database in the National Library of Medicine (http://www.nlm.nih.gov/, accessed on 14 October 2020). For our study, miRNAs and diseases with irregular or incorrect names were deleted. Due to the sparsity of miRNA-disease association types in the miRNA-disease matrix, similarly as [20], we mapped different miRNA precursors into the same mature miRNAs in HMDD v2.0. A summary of the data records in HMDD v2.0 and HMDD v3.2 is presented in Table A1. Figure 1 shows the proportions of associations containing 1, 2, 3, 4 or 5 types.

### 2.2. Similarity Calculation for Diseases and miRNAs

**Figure 2:**
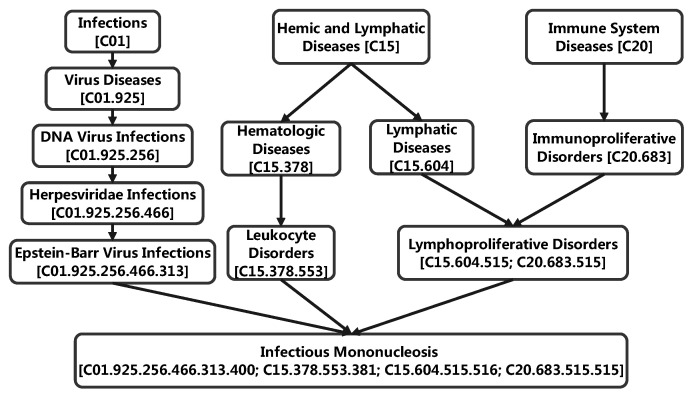
The directed acyclic graph(DAG) of infectious mononucleosis.

The calculation method for the semantic similarity of diseases was proposed by Wang et al. [23]. In the descriptor C of the MeSH database, the relationships among the diseases are represented as directed acyclic graphs (DAGs), which are usually directed from a general disease to a more specific disease. An example of the DAG structure of infectious mononucleosis is shown in Figure 2, where the directionality of the edges is used to find ancestor nodes of infectious mononucleosis, and more distant ancestors contributed less semantically to infectious mononucleosis. For disease *P*, the semantic values DV(P) can be calculated as follows:

(1) $$DV\left(P\right)=\sum_{t\in N_{P}}D_{P}\left(t\right)$$

(2) $$\left\{\begin{array}{cc} D_{P}\left(t\right)=max\;\alpha *D_{P}\left(t\prime \right)|t\prime \in children\left(t\right), & if\;t\neq P\\ D_{P}\left(P\right)=1 \end{array}\right.$$

where $N_{P}$ is the node set including ancestor nodes of *P* and disease *P*. α is the ancestor contribution factor α=0.5 suggested by [23]. since the two diseases with more ancestor nodes are more similar, the semantic similarity between disease *P* and disease *Q* can be calculated as follows.

(3) $$sim\left(P,Q\right)=\frac{\sum_{t\in N_{P}\cap N_{Q}}\left(D_{P}\left(t\right)+D_{Q}\left(t\right)\right)}{DV(P)+DV(Q)}$$

Based on the assumption that similar miRNA precursor sequences will have similar functions, miRNA precursor sequences are effectively used to extract miRNA features or to calculate miRNA similarity in the prediction of miRNA-disease binary association. For example, Li et al. [24] and Ji et al. [25] adopted the 3-mer method to construct miRNAs feature; Che et al. [26] measured the miRNA-miRNA similarity by the Levenshtein distance of miRNA precursor sequences; Zheng et al. [27] calculated an miRNA–miRNA sequence similarity matrix by using a chaos game representation (CGR). In order to make effective use of biological structural information of miRNAs and avoid the recalculation of traditional miRNA function similarity in cross-validation, the miRNAs similarity calculation method proposed by Zheng et al. [27] is adopted in our study where miRNA precursor sequences are mapped to a Euclidean space by an iterative mapping function, and then the region distance of CGRs is calculated to measure the similarity between miRNA and miRNA.

### 2.3. Graph Embedding

A network is a structure composed of nodes and edges, where nodes are connected by edges. Graph embedding is a distributed representation of network structure, which includes node embedding, edge embedding and subgraph embedding. Node embedding can map the discrete nodes in the network to a continuous vector space, and each node has an unique vector representation. Node embedding data after encoding the topological information of the network are very useful inputs relative to machine learning algorithms for downstream tasks, such as node classification and link prediction. In this section, we describe the ideas of Node2vec [28] and GATNE [22], which are used in our prediction as modules.

#### 2.3.1. Node2vec

Node2vec is a graph embedding method, which obtains the nearest neighbor sequence of nodes by a biased random walk. Two hyperparameters *p* and *q* are introduced to balance the depth first search (DFS) and breadth first search (BFS). For the miRNA–miRNA similarity network, given a current miRNA node *v*, let *t* denote its previous miRNA node. The probability of accessing to the next miRNA *x* is calculated as follows:

(4) $$P\left(c_{i+1}=x|c_{i}=v\right)=\left\{\begin{array}{cc} \frac{\alpha_{pq}\left(t,x\right)*w_{vx}}{Z}, & \mathrm{if}\;(v,x)\in E\\ 0, & \mathrm{otherwise} \end{array}\right.$$

(5) $$\alpha_{pq}\left(t,x\right)=\left\{\begin{array}{cc} \frac{1}{p}, & \mathrm{if}\;d_{tx}=0\\ 1, & \mathrm{if}\;d_{tx}=1\\ \frac{1}{q}, & \mathrm{if}\;d_{tx}=2 \end{array}\right.$$

where $\alpha_{pq}\left(t,x\right)$ and $w_{vx}$ are the transition probability and similarity between miRNA *v* and miRNA *x*, respectively; *Z* is the normalized constant. Based on the hypothesis that similar miRNAs are more likely to be associated with the same disease and vice versa, we set p=1,q=2 in this study. Assume that *f* is the mapping function of the miRNA node *u* to the embedding vector. $N_{s}\left(u\right)$ is defined as the set of adjacent nodes of *u*, which is sampled through the sampling strategy *S*. The optimization goal of node2vec is to maximize the co-occurrence probability of the nearby miRNAs for a given current miRNA node [28].

(6) $$max_{f}\sum_{u\in V}\log Pr\left(N_{s}\left(u\right)|f\left(u\right)\right).$$

After embedding, the higher the similarity between two miRNA nodes, the closer the Euclidean distance of their features. The same operation is adopted in disease-disease similarity network.

#### 2.3.2. GATNE

For an attributed network G(V,E,A), $V=\{v_{1},v_{2},...,v_{n}\}$, $E=\{e_{ij}|v_{i},v_{j}\in V\}$ and $A=\{x_{i}|v_{i}\in V$} are the sets of nodes, edges and node features, respectively. If the number of types of the nodes and edges in *G* is larger than 1, *G* is called an attributed multi-layer heterogeneous network (AMHN). $G_{r}=\left\{V,E_{r},A\right\}$ is denoted as a subnetwork of edge type *r*. GATNE [22] is an inductive embedding algorithm on AMHN, composed of three parts: base embedding, edge embedding and node attributes. The core idea of GATNE is to aggregate neighbors from different layers to the current node and then to generate different vector representations for the nodes on each edge type.

Base embedding of a node is shared in different layers. For miRNA nodes in attributed multi-layer miRNA-disease heterogeneous network, the base embedding is obtained through the attributes of miRNAs, and it can be calculated as follows:

(7) $$b_{i}=h_{z}\left(x_{i}\right),$$

where $x_{i}$ is the feature vector of miRNA $v_{i}$ and $h_{z}$ is the transformation function for miRNAs for which its type is defined as *z*.

The edge embedding of attributed multi-layer miRNA-disease heterogeneous network on each relation type is initialized by the transformation function that takes node attributes as input, then the idea of neighbor aggregation in GraphSAGE [29] is adopted to aggregate edge embedding on different levels in single layer. For miRNA node on relation type *r*, we have the following:

(8) $$d_{i,r}^{\left(0\right)}=g_{z,r}\left(x_{i}\right),$$

(9) $$d_{i,r}^{\left(k\right)}=aggregator\left(\left\{d_{i,r}^{\left(k-1\right)},\forall v_{j}\in N_{i,r}\right\}\right),$$

where $g_{z,r}$ is the transformation function, *k* is defined as the *k*th level neighbor of miRNA $v_{i}$ and $N_{i,r}$ is the set of neighbor nodes of $v_{i}$. To take into account the interplays between different relation types, the self-attention mechanism [30] is performed on the concatenate representation constructed by the edge embeddings of nodes from different layers:

(10) $$D_{i}=\left(d_{i,1},d_{i,2},...,d_{i,m}\right),$$

(11) $$a_{i,r}=softmax{\left(w_{r}^{T}\tanh\left(W_{r}D_{i}\right)\right)}^{T},$$

(12) $$u_{i,r}=\alpha_{r}M_{r}D_{r}a_{i,r},$$

where *m* is the number of relation types and $w_{r}$, $W_{r}$ and $M_{r}$ are parameters of relation type *r*, which can be trained by parameter optimization.

The embedding representation of miRNA node $v_{i}$ on relation type *r* is calculated as follows.

(13) $$v_{i,r}=\beta_{r}D_{z}^{T}x_{i}+b_{i}+u_{i,r}.$$

The same embedding operations are adopted on disease nodes.

In parameter optimization, the meta-path-based random walk [31] and skip gram [32] on the heterogeneous network and negative sampling are adopted to optimize the parameters of GATNE. The objective function is as follows:

(14) $$\begin{array}{cc} E\; & =-logP_{\theta }\left(\left\{v_{j}|v_{j}\in C\right\}|v_{i}\right)\\ \; & =-log\sigma \left(c_{j}^{T}\cdot v_{i,j}\right)-\sum_{l=1}^{L}E_{v_{k}∼P_{t}\left(v\right)}\left[log\sigma \left(-c_{k}^{T}\cdot v_{i,r}\right)\right], \end{array}$$

where the context *C* of path $P=(v_{p1,...,pt})$ is generated by meta-path-based random walk and $c_{k}$ is the context embedding of node $v_{k}$, which is a negative sample randomly selected from distribution $P_{t}\left(v\right)$, σ(x)=1/(1+exp(−x)). θ represents all parameters in GATNE.

### 2.4. mDLinker

**Figure 3:**
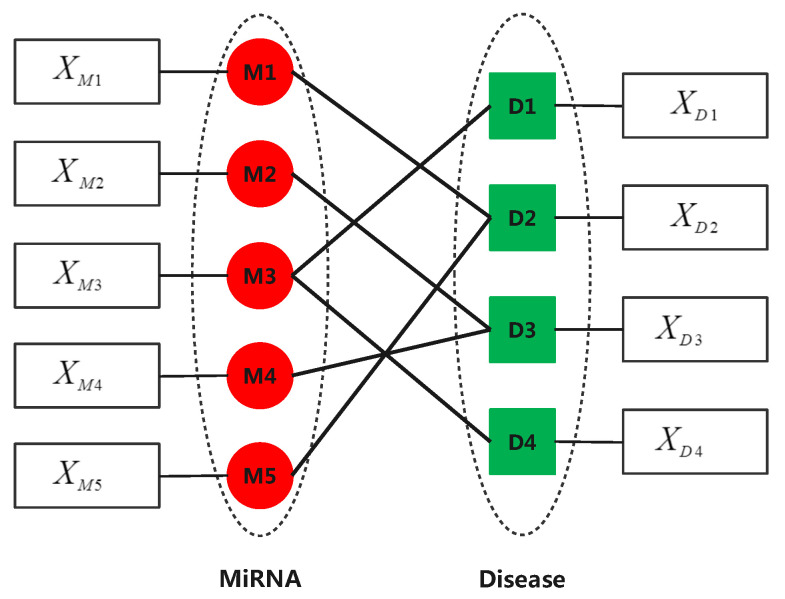
A single layer in our attributed multi-layer miRNA-disease heterogeneous network, where $X_{Mi}$ and $X_{Dj}$ represent the feature vector of miRNA Mi and that of disease Dj, respectively.

In this paper, we propose a novel algorithm (named mDLinker) to predict multiple association types between miRNAs and diseases through a multi-layer heterogeneous network embedding technique. The first step of mDLinker is to calculate miRNA–miRNA similarities based on the miRNAs’ sequences and calculate disease–disease similarities based on the DAG structure of the diseases. Denote the miRNA–miRNA similarity network as $G_{M}=\left(V_{M},E_{M}\right)$ with the weighted adjacency matrix $A_{M}\in R^{m\times m}$, where $A_{M}\left(i,j\right)$ is the similarity between miRNAs *i* and *j*, $V_{M}=\left\{1,2,...,m\right\}$ is the set of miRNA nodes and $E_{M}$ is the set of edges. The second step of mDLinker is to obtain the feature matrix of miRNA nodes $X_{M}\in R^{m\times NE}$ by Node2vec, where NE represents the feature dimension of miRNA nodes after embedding. Similarly, we obtained the disease–disease similarity network $G_{D}=\left(V_{D},E_{D}\right)$ with the weighted adjacency matrix $A_{D}\in R^{n\times n}$, $V_{d}=\left\{1,2,...,n\right\}$ and determine the feature matrix $X_{D}\in R^{n\times NE}$ through Node2vec. Then, we constructed an attributed multi-layer miRNA-disease heterogeneous network (AMH-MD), where the only difference between each layer of AMH-MD is the type of association. One of these layers is shown in Figure 3. In AMH-MD, the topologies of all single miRNA-disease association and the relationships between different types of association, as well as the feature of nodes, are well described.

**Figure 4:**
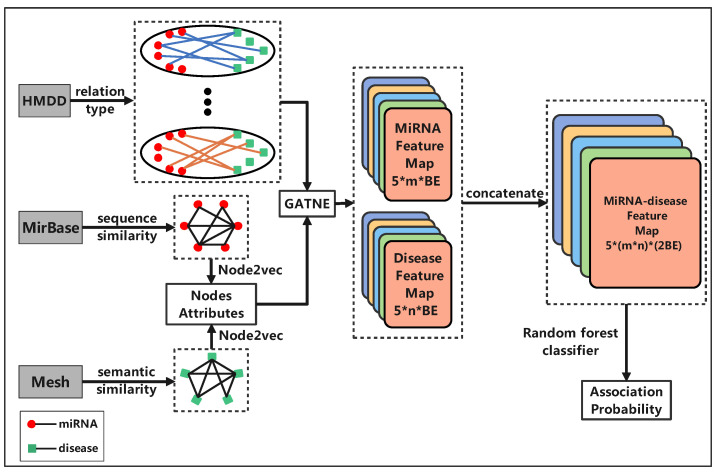
An illustration of predicting multiple types of miRNA-disease associations by mDLinker. There are *m* miRNAs, *n* diseases and five layers in the network representing the five types of associations. BE is the dimension number of the miRNA vector representation and also the dimension number of the disease vector representation embedded by GANTE.

Let MD-P denote an miRNA-disease pair that is known with an association, but the association is not specified with any type; and let MD-T denote an miRNA-disease-type triple, meaning the type of the association between the miRNA and disease is specified. Given the complicated correlations between the MD-Ts, for each association type, the graph embedding technique GATNE is applied by our method mDLinker to aggregate topology information from different layers to the current layer such that the embedding representation of the miRNAs and diseases on each layer can be characterized. Then, we train a machine learning classifier on these experimentally confirmed MD-Ts and a set of selected negative samples, where the feature vectors of MD-Ts are obtained by concatenating the two vector representations of corresponding diseases and miRNAs. The algorithm flow diagram is shown in Figure 4.

## 3. Experiments and Results

### 3.1. Experimental Setting

In order to validate the performance of mDLinker, we adopted two kinds of experiments, termed CV-Type and CV-Triple, as similarly used by Huang et al. [21] for a fair comparison.

By CV-Type, we randomly divided the confirmed MD-P instances into 10 equal parts, one of which was reserved as a test set and the others are used as the training set. In both the test set and training set, experimentally verified miRNA-disease-type triples are regarded as the positive samples, while the unconfirmed or non-existent MD-Ts are served as negative samples. For each MD-P in the test set, the association probabilities of all types of MD-T are predicted. If the top one ranked MD-T is confirmed by HMDD, the corresponding MD-P is predicted correctly. The purpose of the CV-Type is to demonstrate the ability of exploring the most reliable MD-T from the corresponding MD-P. Precision, recall and f1 of top one are calculated in CV-Type.

In CV-Triple, we randomly divided all MD-T instances into 10 equal parts, one part reserved as a test set, and the others form the training set. Due to the lack of MD-T negative samples provided by biologists, we constructed a set $N_{sample}$ of negative samples from the unconfirmed or non-existent MD-T by a strategy similar to [33,34]. For relation type *r*, we calculated an average representation feature $f_{avg,r}$ of all positive samples in the training set. The Euclidean distances from $f_{avg,r}$ to all unconfirmed or non-existent MD-T are measured, where the average Euclidean distance is denoted as $dis_{r}$. For MD-T *i*, if the distance $dis_{r,i}$ from $f_{avg,r}$ to *i* is greater than $dis_{r}$, MD-T *i* can be regarded as a more reliable negative sample on relation type *r*. In this experiment, we randomly selected negative samples equal to the number of positive samples on each edge type from $N_{sample}$. Three performance indicators of AUPR, AUC and f1-measure are calculated to confirm the effectiveness of mDLinker.

### 3.2. **Performance by Different Classifiers**

We investigated the performance of different classifiers on predicting the potential miRNA-disease-type triples under the setting CV-Type. Several classifiers were attempted, including decision tree, naive Bayes, logistic regression, KNN, SVM and random forest. The performances are shown in Table 1. As an ensemble classifier, random forest achieved the best performance.

### 3.3. Performance Comparison with the State of the Art

Several methods have been proposed to predict the types of miRNA-disease associations. We compared the performance of our mDLinker with two newest algorithms TFAI [21] and TDRC [21] on the same data set used in our paper from HMDD v3.2. NLPMMDA [20] can effectively predict each association type, respectively, but it only models the binary association on each type of miRNA-disease association. On the one hand, according to the comparison results of Huang et al. [21], TDRC algorithm has the best performance compared to the other methods, and it is significantly superior to NLPMMDA. On the other hand, we cannot run NLPMMDA on our data set because the authors of NLPMMDA did not provide the source code of it. Thus, we did not perform the comparison between mDLinker and NLPMMDA in our study. The parameters in TFAI and TDRC are set as suggested by the original paper. From Table 2 and Table 3, one can observe that mDLinker consistently outperforms TFAI and TDRC under all evaluation criteria. By the ranking-based evaluation (Table 2), mDLinker, TFAI and TDRC achieved top one f1 of 0.5649, 0.4832 and 0.5071, respectively. This suggests that utilizing non-linear relationships between miRNA nodes and disease nodes has greatly contributed to the high performance of identifying potential MD-T from MD-P. Under the setting CV-Triple, the most significant improvement is made again by mDLinker. In particular, the scores of F1, AUPR and AUC are improved by 4.99%, 5.15% and 6.41%, respectively, in comparison with the previous best model TDRC.

### 3.4. Sensitivity Study on Parameters

Sensitivity analyses were conducted on three hyper-parameters: NE (the attribute dimension of miRNA nodes and disease nodes), DE (the dimension of edge embedding) and BE (the dimension of base embedding). These three hyper-parameters determine the framework of mDLinker and affect the performance of prediction.

**Figure 5:**
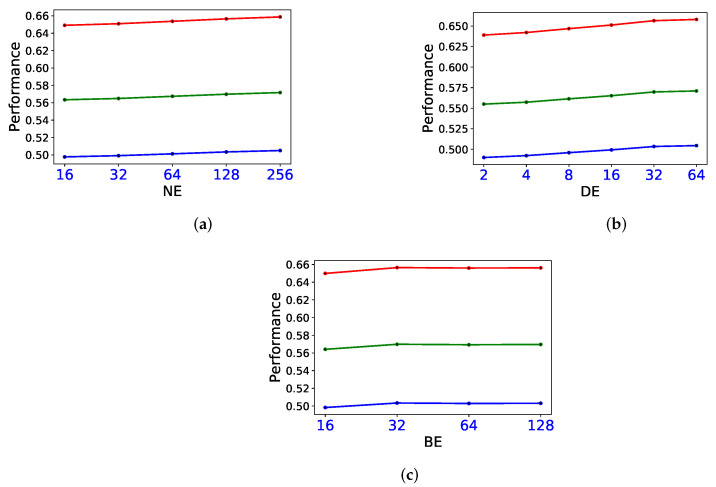
Sensitivity analysis on different parameter settings, including dimension of node attribute (**a**), dimension of edge embedding (**b**) and dimension of base embedding (**c**), where the red polyline represents Top-1 precision, the green polyline represents Top-1 F1 and the blue polyline represents Top-1 recall.

Figure 5 shows the performance when different hyper-parameter settings were tested under CV-Type. NE was ranged in {16,32,64,128,256}. It can be observed that when NE becomes larger, the performance trends better. Therefore, we set NE=128 to prevent overfitting. DE and BE are searched in the ranges {2,4,8,16,32,64} and {16,32,64,128}. The best and most stable performance is achieved when DE=32 and BE=32.

### 3.5. Case Study: miRNA-Disease Association Types Predicted beyond the HMDD Databases

**Figure 6:**
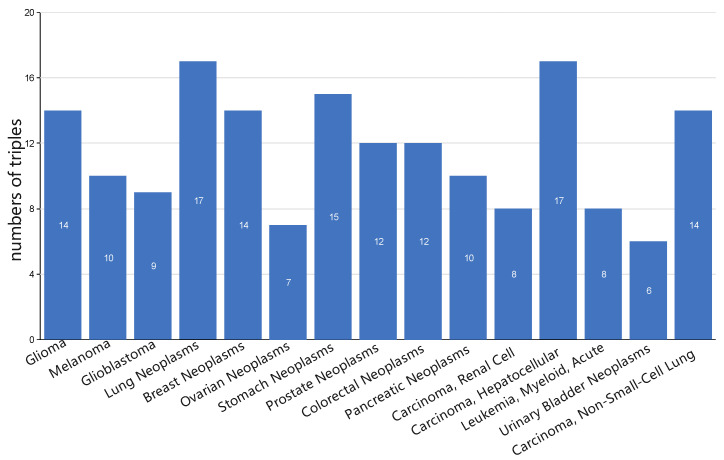
Case study on 15 diseases unique in HMDD v3.2. The training model is based on the miRNA-disease-type triples recorded in HMDD v2.0. Numbers of predicted miRNA-disease associations, which are confirmed by HMDD v3.2, are highlighted in the middle of the bars.

The database HMDD v3.2 has recorded much more miRNA-disease associations than its early version 2.0. We conducted a case study to observe whether the newly recorded miRNA-disease associations and their types in HMDD v3.2 can be predicted by a mDLinker model established from HMDD v2.0. For this case study, we used the disease name mapping table provided by Huang et al. [35] to map disease names from HMDD v2.0 to HMDD v3.2, and we selected 15 unique disease names in HMDD v3.2 that each have at least 20 miRNA-disease-type triples (i.e., at least 20 miRNA-disease associations of different types) as test cases. Node2vec and GATNE are sampled via biased random walk and the meta-path-based random walk, respectively. In order to eliminate the error caused by sampling, we ran it 50 times and considered the top 20 MD-Ts with the highest consensus frequency as the prediction result. Figure 6 shows the detailed prediction performance by mDLinker on the 15 diseases. We can observe that many of the predicted associations are actual associations recorded in HMDD v3.2, especially for lung neoplasms and hepatocellular carcinoma, where 85% of the top 20 predicted associations are actual associations confirmed in HMDD v3.2.

Our second case study is to use all the associations and the association type information stored at HMDD v3.2 to train our prediction model and then to apply this model to predict currently unknown miRNA-disease associations and their types. We run the model 50 times. Each time, from the miRNA-disease-type triples that are not recorded in HMDD v3.2, we stored those triples with the highest association probability predicted by our model. Over 50 times, the top 10 predicted associations with the most consensus in the stored top lists were regarded as the most likely miRNA-disease associations with a specific type. For example, an miRNA-target association type between mir-224 and Gastric Neoplasms predicted by our method mDLinker was confirmed by Fang et al. [36], and mir-224-5p negatively regulates OPCML in gastric cancer tissues; the abnormal expression of miR-148a in prostate cancer predicted by MDLinker is consistent with the findings by Fujita et al. [37] that the expression level of miR-148a in PC3 and DU145 hormone-resistant prostate cancer cells is lower than that in PrEC normal prostate epithelial cells. More details of these predicted associations are presented in Table 4. Although none of them are currently recorded in HMDD v3.2, all of these predictions can be confirmed by the literature works from NCBI (see the PMIDs at the fourth column of the table).

## 4. Conclusions

It is well known that there is a strong correlation between disease and miRNA. miRNA can be used as a diagnostic marker and therapeutic target for disease. Unless evidence of miRNA-disease association is confirmed, it is not sufficient to understand pathology at the molecular level of miRNA. In this study, we have proposed an innovative algorithm (mDLinker) based on embedding techniques and machine learning ideas in order to predict the heterogeneous types of miRNA-disease associations and showed the significance and usefulness of utilizing non-linear relationships in uncovering potential miRNA-disease-type triples. By mDLinker, the miRNAs and diseases attributes are obtained through Node2vec to embed the miRNA sequence information and DAG structure information of the diseases, respectively. All the nodes in AMH-MD are projected into a vector on each association by the powerful embedding technique GATNE. In order to calculate the probability of each association type between an miRNA and a disease, the random forest classifier is adopted. Compared with the state-of-the-art algorithms, mDLinker achieves significantly better performance in the systematical experiments. Furthermore, it performs excellent in the prediction of currently unknown associations in the HMDD v3.2, as demonstrated in our case studies. The mDLinker is shown to be a reliable model in exploring multiple relationships between miRNAs and diseases, and it is a useful tool in enhancing understanding of the molecular basis of disease formation.

## Figures and Tables

**Table 1:** Ten-fold cross validation performance in CV-Type by different classifiers based on HMDD v3.2.

Method	Top-1 Precision	Top-1 Recall	Top-1 F1
Decision tree	0.4203	0.3224	0.3649
Naive Bayes	0.4748	0.3640	0.4121
Logistic Regression	0.4960	0.3803	0.4305
KNN	0.6001	0.4602	0.5209
SVM	0.5706	0.4357	0.4952
Random Forest	0.6509	0.4991	0.5649

**Table 2:** Performance comparison among TFAI, TDRC and mDLinker under the setting CV-Type.

Method	Top-1 Precision	Top-1 Recall	Top-1 F1
TFAI	0.5874	0.4501	0.4832
TDRC	0.6116	0.4686	0.5071
mDLinker	0.6509	0.4991	0.5649

**Table 3:** Performance comparison among TFAI, TDRC and mDLinker under the setting CV-Triple.

Method	AUPR	AUC	F1
TFAI	0.9261	0.912	0.8559
TDRC	0.93	0.9222	0.865
mDLinker	0.9799	0.9737	0.9291

**Table 4:** miRNA-disease associations predicted by our model trained on HMDD v3.2 with their association types and their supporting literature from NCBI.

MiRNA	Disease	Type	PMID	Experimental Methods	Description
hsa-mir-224	Gastric Neoplasms	Target	32359894	RT-qPCR; Western blot analysis	OPCML is negatively regulated by miR-224-5p in Gastric cancer tissues.
hsa-mir-193a	Carcinoma, Hepatocellular	Target	30710422	qRT-PCR; Dual luciferase reporter assay; RNA pull-down assay	miR-193a-5p inhibits the growth of hepatocellular carcinoma by targeting SPOCK1.
hsa-mir-31	Gastric Neoplasms	Target	30677405	Silico analysis; Dual luciferase reporter assay	Zeste homolog 2 (ZH2) is the potential target of miR-31 in AGS cells to inhibit Gastric cancer.
hsa-mir-218-1	Carcinoma, Hepatocellular	Target	30003726	Fluorescence protein analysis; RT-qPCR; Western blotting	miR-218 suppresses the growth of hepatocellular carcinoma by inhibiting the expression of proto-oncogene Bmi-1.
hsa-mir-148a	Prostate Neoplasms	Tissue	20406806	the trypan blue; dye exclusion assay	miR-148a expression levels are lower in PC3 and DU145 hormone-refractory prostate cancer cells than PrEC normal human prostate epithelial cells.
hsa-mir-218-1	Breast Neoplasms	Target	29378184	RT-qPCR analysis; Luciferase reporter assay; Cancer biostatistical analysis	miR-218 regulates breast cancer progression by targeting Lamins.
hsa-let-7	Carcinoma, Hepatocellular	Target	27821157	MTT assay; western blot; immunofluorescence; luciferase-reporter assay	Let-7 inhibits the self-renewal of stem cell-like cells by regulating Wnt signaling pathwayand EMT.
hsa-mir-193a	Colorectal Carcinoma	Target	29104111	qRT-PCR; Western bolt analysis	MiR-193a-3p plays a tumor suppressive role by targeting KRAS in colorectal adenocarcinoma patients.
hsa-mir-200c	Carcinoma, Cervical	Target	27693631	Luciferase reporter; qRT-PCR assays	Disrupting MALAT1/miR-200c sponge decreases invasion and migration in endometrioid endometrial carcinoma.
hsa-mir-34c	Ovarian Neoplasms	Target	32308421	qRT-PCR; MTT; Western blot assays; Immunoprecipitation; Flow cytometry analysis	miR-34c targets MET to improve the Anti-Tumor effect of Cisplatin on ovarian cancer.

## Data Availability

Not applicable.

## Appendix A

**Table A1:** Details of the two data sets in this work: *a* represents miRNA-disease pair association, and *b* represents miRNA-disease-type triple.

HMDD	miRNA	Disease	MD-P ^*a*^	MD-T ^*b*^				
				Type-1	Type-2	Type-3	Type-4	Type-5
v2.0	321	168	1506	355	215	441	676	0
v3.2	695	445	12,495	1578	519	3300	5822	5079
